# A Maxillary Second Molar with Two Separate Palatal Roots: A Case Report

**Published:** 2013-06

**Authors:** E Fakhari, A Shokraneh

**Affiliations:** a Dept. of Periodontics, School of Dentistry, Golestan University of Medical Sciences, Gorgan, Iran; b Dept. of Endodontics, School of Dentistry, Isfahan University of Medical Sciences, Isfahan, Iran

**Keywords:** Maxillary Molars Root Canal Variation

## Abstract

Variations of dental root canals were reported by different authors. One of the rare variations is the presence of two separate palatal roots of maxillary molars, especially second maxillary molars. This case study reported a maxillary second molar with two separate palatal roots and a palatal bifurcation which was found during the periodontal flap surgery. Although these variations are rare, awareness of their presence would help in successful periodontal and endodontic treatment.

## Introduction

One of the important reasons of the failure in the endodontic treatment is the presence of the microorganisms remained by the incomplete instrumentation, inadequate cleaning, insufficient canal obturation and the presence of untreated canals [1].

The maxillary second molars resemble the maxillary first molars anatomically. The distinctive morphologic feature is that the three roots are united closer and are sometimes fused. Also, they are generally shorter than the roots of the first molars and are that much curved. The second molars usually have only one canal in each root. Four canals are less likely seen in the second molars than in the first molar. The three main orifices usually form a flat triangle and sometimes they produce almost a straight line. In general, the canals are closer mesially to each other than they are in maxillary first molars. The floor of the pulp chamber is markedly convex, which gives the canal orifices a slight funnel shape [2].

There are few studies about the anatomic variations of maxillary second molars. Variations mostly could be seen in the mesiobuccal roots [3] and particularly the mesiobuccal canal in 30-80% of the cases [4]. However, the frequency of reports on two palatal roots with two canals is low [5-9]. Peikoff et al. reported a 1.4% incidence of four separate roots and four separate canals including two palatal roots in 520 maxillary second molars [10].

Alani AH [11] encountered four roots in the second maxillary molars in one patient. Filho *et al.* [7] carried out an *in vitro* study of two maxillary second molars with four canals and two different palatal roots. Barbizam et al. [8] reported a study of a second maxillary molar with four canals in four distinct roots. Moreover, Benenati et al. presented a clinical case of a second maxillary molar with two palatal roots [9].

A double palatal root is not easy to detect clinically, as the extra root canal usually is superimposed by buccal root canals. This issue may complicate root canal treatment and increase the failure rate. A number of reports [12-16] have addressed the morphological variations of the root canal system of palatal root of the maxillary molars.

This article presented a maxillary second molar with two separate palatal roots which was found during periodontal flap surgery.

## Case Report

A 45-year old man was referred to the department of periodontics for periodontal treatment. The chief complaints of the patient were bleeding on brushing teeth and a dull pain in the maxillary left quadrant. Gingival redness, bleeding on probing and pocket depth of 6 mm on palatal surface of molars was found in clinical examination. Pocket depths on buccal, mesial and distal surfaces were 4-5 mm. Besides, there was a generalized gingival inflammation with probing depth of 3-4mm in more than 30% of areas. Vitality test was positive for both of maxillary left molars and there was no percussion sensitivity. There were buccal furcation involvement (grade ΙΙΙ) and mobility (grade II).

**Figure 1a:**
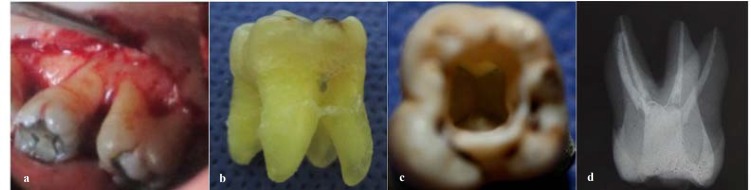
Palatal furcation in the maxillary left second molar **b **Extracted maxillary left second molar(palatal aspect) **c **Access cavity preparation of maxillary left second molar **d **Root canal treatment of maxillary left second molar with four separate roots

Periodontal diagnosis was generalized moderate chronic periodontitis. Phase Ι in periodontal treatment, consisting the scaling and root planning, was performed for the patient in two sessions. After 6 weeks, the clinical signs of the gingival inflammation were greatly subsided and the plaque score was less than 10%. The pocket depth changed to 4 mm in the maxillary molars but there was persistent inflammation in the maxillary second molar area. So the conventional flap was elevated and after debridement, a palatal furcation could be seen in the maxillary left second molar from palatal aspect (Figure 1a).

There was grade ΙΙΙ palatal furcation involvement and it had no antagonist tooth. So the prognosis of the tooth was assumed hopeless and it was extracted during the periodontal surgery. As it is displayed in the figure 1b, the tooth had two separate palatal roots with one canal in each root (Figure 1b).

The access cavity of the maxillary second molar is usually triangular in shape. The base and the top of this access cavity is the buccal and palatal aspect of the tooth respectively without oblique ridge involvement. In the current case, to obtain a straight line access to the canals, the access cavity was prepared in trapezoidal shape and involved the oblique ridge. The pulp chamber of this tooth was broader in the palatal area and like other teeth, represented the whole crown shape (Figure 1c).

Root canal treatment ‌was performed and the working length was determined and the root canals were cleaned and shaped using Gates Glidden drills, stainless steel hand K-files, and Pro Taper nickel-titanium rotary instrumentation (Dentsply Maillefer, Switzerland) under the copious irrigation with 2.5% NaOCl solution. The root canals were dried and were obturated using a cold lateral condensation technique with gutta-percha cones and a resin-based sealer, AH-plus (Dentsply Maillefer, Switzerland). The final radiograph revealed two separate roots with one canal in each root (Figure 1d).

## Discussion

This report presents a variation of the maxillary second molar which dental practitioners do not frequently consider in practice. A maxillary second molar with palatal bifurcation was found during a periodontal flap surgery. It was extracted because of the periodontal problem and the absence of antagonist tooth. Endodontic treatment determined two separate palatal roots with one canal in each root. Up to now, this variant with four separate roots and four separate canals, including two palatal roots, was the least frequent abnormality, with incidence ranging from 1.47 to 2.1% [9, 15].

Kim et al. [17] demonstrated that the palatal roots had one root with one canal among 150 maxillary first molars. Cleghorn et al. [4] reported the incidences of palatal roots with a single canal and a single foramen to be 99% and 98.8% respectively. The unusual anatomy of the maxillary second molar is difficult to diagnose because of its posterior location. The superposition of the anatomical structures on the radiographs of this region may fail the diagnosis of a second palatal root canal. Taking several radiographs from different angles helped us overcome the superimpositions; therefore we could detect this rare abnormality [18]. Visualization of the pulp chamber floor and the canals with the endodontic microscope; the exploration of the canal walls with the pre-curved hand files and the use of the electronic apex locator could also be helpful [19]. Detecting a palatogingival groove on the lingual surface of the crown and the root can be another possible sign of two separate palatal canals [20]. Studies have shown that only three dimensional radiographs could grant accurate information about the location of both palatal canals either within one common root or in the two separate roots [19].

Although these variations are not common, it is important for clinicians to be aware of the unusual root morphologies and canal configurations. This can give support to the clinicians in the diagnosis and endodontic treatment of the maxillary molars to avoid incomplete root canal preparation and subsequently, to decrease the treatment failure.

## Conclusion

For a successful endodontic and periodontal treatment, it is important to keep in mind that there is a chance of encountering a maxillary second molar with two palatal roots and palatal bifurcation.
